# Coexisting PTEN and SDHB Mutations in a Pediatric Patient with PTEN Hamartoma Tumor Syndrome: a case report

**DOI:** 10.3389/fped.2026.1909252

**Published:** 2026-07-28

**Authors:** Yaqin Zhang, Sooyeon Lee, Justin P. Annes, Jie Zhang, Taicheng Zhou, Yuan Qian, Xingli Deng

**Affiliations:** 1 Department of Reproductive Genetics, Pu'er People's Hospital, Pu'er, China; 2 Department of Medicine, Division of Endocrinology, Stanford University, Stanford, CA, United States; 3 Stanford ChEM-H and Endocrine Oncology, Stanford University Cancer Institute, Stanford, CA, United States; 4 Genetics Department, The First People's Hospital of Yunnan Province, Kunming, China; 5 Central Lab, Clinical Diagnosis and Treatment Center for Precision Medicine, The Affiliated Hospital of Yunnan University/Second People's Hospital of Yunnan Province, Kunming, China; 6 Department of Neurosurgery, The First Affiliated Hospital of Kunming Medical University, Kunming, China

**Keywords:** macrocephaly, multigenic interaction, precision medicine, PTEN hamartoma tumor syndrome, *PTEN mutation*, *SDHB variant*

## Abstract

**Background:**

Phenotypic variability in PTEN hamartoma tumor syndrome (PHTS) remains incompletely understood, and the contribution of additional rare genetic variants has rarely been functionally investigated.

**Case presentation:**

We report a 9-year-old girl with macrocephaly, gastrointestinal manifestations, hypogammaglobulinemia, and lymphocyte abnormalities. Thyroid function and biochemical screening for SDHB-associated tumors were normal. Trio-based whole-exome sequencing identified a *de novo* pathogenic *PTEN* variant, c.464A > G (p.Tyr155Cys), together with a *de novo SDHB* variant, c.719T > C (p.Leu240Pro). Functional studies showed an increased succinate-to-fumarate ratio and reduced succinate dehydrogenase activity, consistent with impaired mitochondrial complex II function. Structural modeling further suggested that the p.Leu240Pro substitution may reduce SDHB protein stability. Functional evidence fulfilled ACMG/AMP PS3 criteria and supported the proposed reinterpretation of the *SDHB* variant from a variant of uncertain significance to likely pathogenic using the OddsPath framework.

**Conclusions:**

This case expands the molecular spectrum of PHTS by describing the coexistence of pathogenic *PTEN* and functionally supported *SDHB* variants. Although the phenotype is largely attributable to PHTS, the potential contribution of SDHB dysfunction remains speculative and warrants further investigation. These findings underscore the importance of functional variant interpretation and genotype-informed longitudinal surveillance in rare genetic disorders.

## 1 Introduction

PHTS is a group of rare genetic disorders caused by germline pathogenic variants in the PTEN tumor suppressor gene, characterized primarily by multi-system hamartomas and an increased susceptibility to benign and malignant tumors (1). The disease spectrum is broad, encompassing Cowden syndrome (CS), Bannayan-Riley-Ruvalcaba syndrome (BRRS), PTEN-related Proteus syndrome, and Proteus-like syndrome, which exhibit significant overlap in their clinical manifestations. Research indicates that the incidence of PHTS is approximately 1/7,500, a figure significantly higher than historical estimates, suggesting that the disease may be severely underdiagnosed or insufficiently diagnosed in the general population (2). Furthermore, the clinical phenotype of PHTS is highly heterogeneous; beyond the defined cancer risk, it has been recognized to be one of the common causes of monogenic autism spectrum disorder (3). Although PHTS is relatively rare in the pediatric population, a high index of suspicion for PHTS should be maintained in children presenting with macrocephaly accompanied by autism spectrum disorder or intellectual disability, as these neurodevelopmental abnormalities are often critical clues for the early identification of the syndrome (4).

All syndromes associated with PHTS arise from germline mutations in the *PTEN* gene, encompassing point mutations, deletions, insertions, and extensive genomic rearrangements. These genetic alterations lead to a loss or reduction of PTEN function, which in turn results in the aberrant activation of the PI3K/AKT signaling pathway, thereby facilitating uncontrolled cellular proliferation and the formation of hamartomas. Despite the shared genetic etiology, the clinical manifestations of these syndromes exhibit considerable variability. This phenotypic heterogeneity may be attributed to differences in mutation types, variable penetrance, and the influence of genetic modifiers (5, 6). For example, certain mutations are more frequently associated with neurological involvement, whereas others predominantly impact the skin or gastrointestinal system (7).

The inheritance pattern for PHTS is autosomal dominant. About 10%–50% of people with CS have an affected parent, while the majority of cases happen infrequently (8, 9). The actual percentage of sporadic vs. familial cases is unknown, though, because of possible underdiagnosis or inconsistent clinical recognition. CS is one of the most prevalent phenotypic forms of PHTS among its clinical manifestations. The breast, thyroid, endometrium, and kidneys are the organ systems most commonly affected by the numerous benign and malignant tumors that people with CS usually develop (10, 11). Research indicates that almost 80% of individuals with CS had pathogenic *PTEN* mutations, which markedly elevate the lifetime risk of developing various malignancies (12). Furthermore, it has been found that between 10.7% and 47.6% of afflicted individuals have *de novo PTEN* mutations, suggesting that PHTS can develop even in the absence of a positive family history (5).

A multidisciplinary approach is necessary for the diagnosis and treatment of PHTS. Early identification and routine surveillance are crucial for the prevention and prompt detection of related malignancies because of the wide range and complexity of its clinical manifestations (6, 13). In clinical practice, the diagnosis of PHTS is mainly made using molecular confirmation of *PTEN* germline mutations along with distinctive clinical features.

Apart from the elevated risk of multisystem tumors, one of the most prevalent clinical manifestations is macrocephaly, which is especially noticeable in patients with BRRS and CS. Significant head enlargement can occasionally be seen as early as childhood (14). Additionally common are cutaneous manifestations, such as mucocutaneous papillomas and trichilemmomas (15). Some people may experience neurological abnormalities, and some patients may develop dysplastic gangliocytoma of the cerebellum (Lhermitte–Duclos disease), which is distinguished by a characteristic “tiger-striped” pattern on magnetic resonance imaging (MRI) (16). Other lesions like esophageal glycogenic acanthosis and gastric polyps may be present in the gastrointestinal system in addition to hamartomatous polyps of the intestine. These lesions may affect digestive function and cause symptoms like dyspepsia, abdominal pain, and hematochezia (17, 18).

When a germline pathogenic variant in the *PTEN* gene is found in the patient, the diagnosis of PHTS can be genetically confirmed (10). Whole-exome sequencing (WES) or whole-genome sequencing (WGS) may be used to find rare variants or structural abnormalities that conventional methods might overlook in people with strong clinical suspicion but negative results from traditional genetic testing (19). Furthermore, immunohistochemical examination of PTEN protein expression in tumor tissues can be a useful supplementary technique to help confirm the diagnosis in suspected cases (20).

In terms of therapy, endoscopic or surgical resection is recommended for pertinent tumors or severe clinical symptoms (21). Pharmacologically, mTOR inhibitors are thought to have potential therapeutic efficacy because PTEN inactivation causes the PI3K/AKT/mTOR signaling pathway to become hyperactivated (22, 23). Nevertheless, there are still issues with pharmacological treatments, such as side effects, long-term safety issues, and inter-individual differences in therapeutic response, which emphasizes the need for more research (24).

We present a case study of a 9-year-old patient exhibiting coexisting heterozygous variants in the *PTEN* (c.464A > G, p.Tyr155Cys) and *SDHB* (c.719T > C, p.Leu240Pro) genes. The patient began experiencing abdominal pain at the age of 8, with symptoms persisting for over six months. The patient's medical history includes diagnoses of macrocephaly, colonic polyps, gastritis, and hypogammaglobulinemia. Upon physical examination, the patient's skin was intact, showing no signs of rash, jaundice, petechiae, or ecchymosis. Additionally, the oral mucosa was moist, and skin elasticity was within normal limits. Trio-based WES was conducted, identifying coexisting *de novo* variants in *PTEN* and *SDHB*. This case may further expand the reported clinical and molecular spectrum of PHTS.

## 2 Case presentation

The 9-year-old girl patient measures 143 cm in height, 37 kg in weight, and has a head circumference of 56.5 cm. She was born with a birth weight of 2.5 kg at 34 weeks of gestation. During infancy, she had poor swallowing function and frequently experienced choking during feeding. Other than these findings, the physical examination was unremarkable. She responded fluently and was cooperative and attentive. A review of the family history did not reveal a pattern suggestive of a hereditary disorder. Her parents have a healthy first child, a son, and the proband is their second child, conceived when the mother and father were 38 and 39 years old, respectively. Non-paternity was excluded by genetic testing, supporting biological parentage.

**Figure 1:**
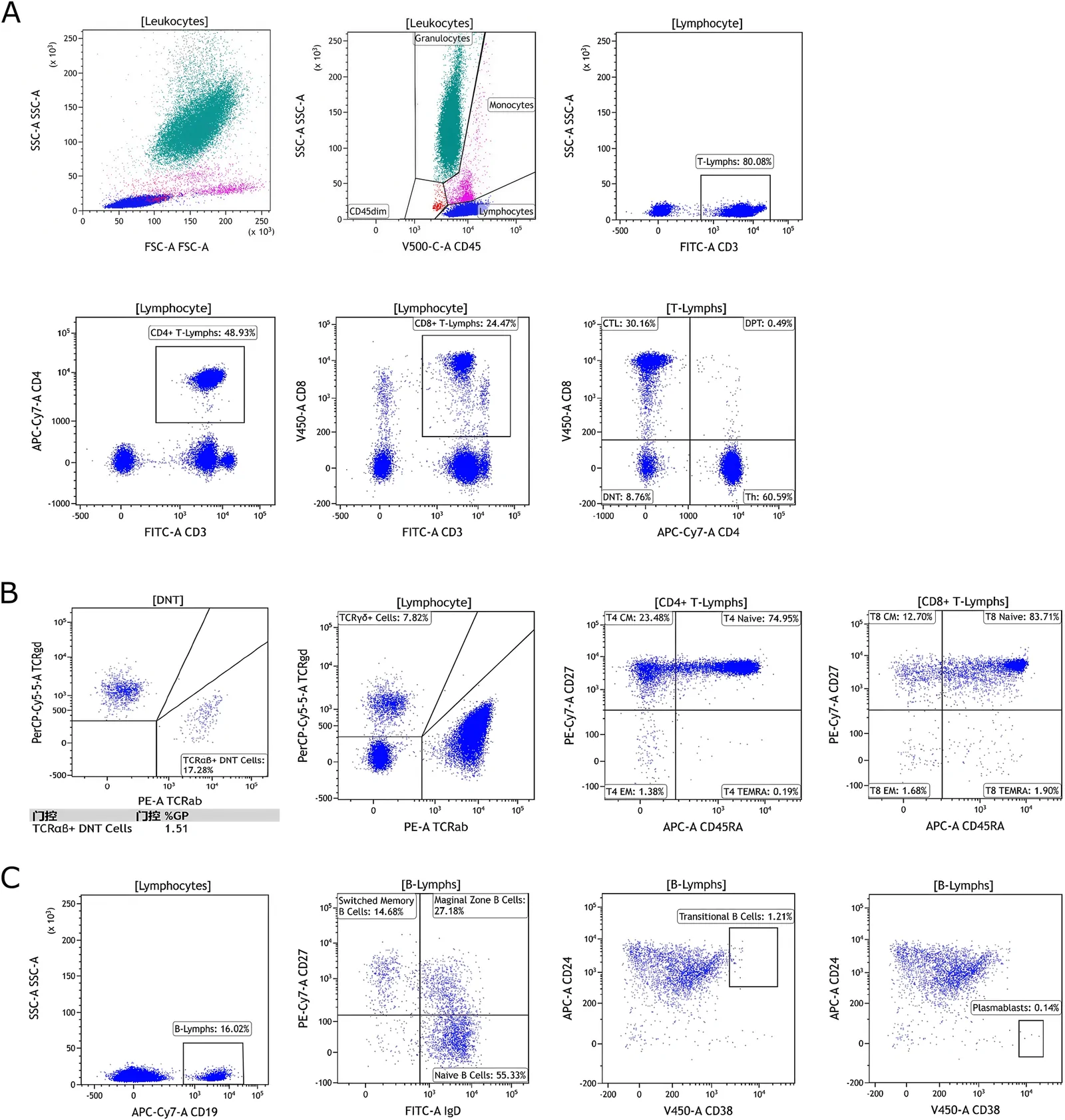
Detailed lymphocyte immunophenotyping. **(A)** T cell subsets **(B)** Detailed T cell subpopulations **(C)** Detailed B cell subpopulations.

The patient, aged 8, exhibited intermittent abdominal pain lasting roughly six months. Colonoscopy showed that the terminal ileum and rectal mucosa were inflamed. Histopathological analysis did not reveal characteristics typical of inflammatory bowel disease, nor were tumors or granulomatous lesions indicative of tuberculosis detected. Detailed lymphocyte immunophenotyping showed that there were more total T cells (80.08%), CD4⁺ T cells (48.93%), naïve CD4⁺ T cells (74.95%), naïve CD8⁺ T cells (83.71%), and marginal zone B cells (27.18%). On the other hand, there were fewer CD4⁺ effector memory T cells (1.38%), CD8⁺ central memory T cells (12.70%), CD8⁺ effector memory T cells (1.68%), transitional B cells (1.21%), and plasmablasts (0.14%) (Figure 1). All immunophenotyping results were interpreted according to age-adjusted institutional reference intervals.

**Figure 2:**
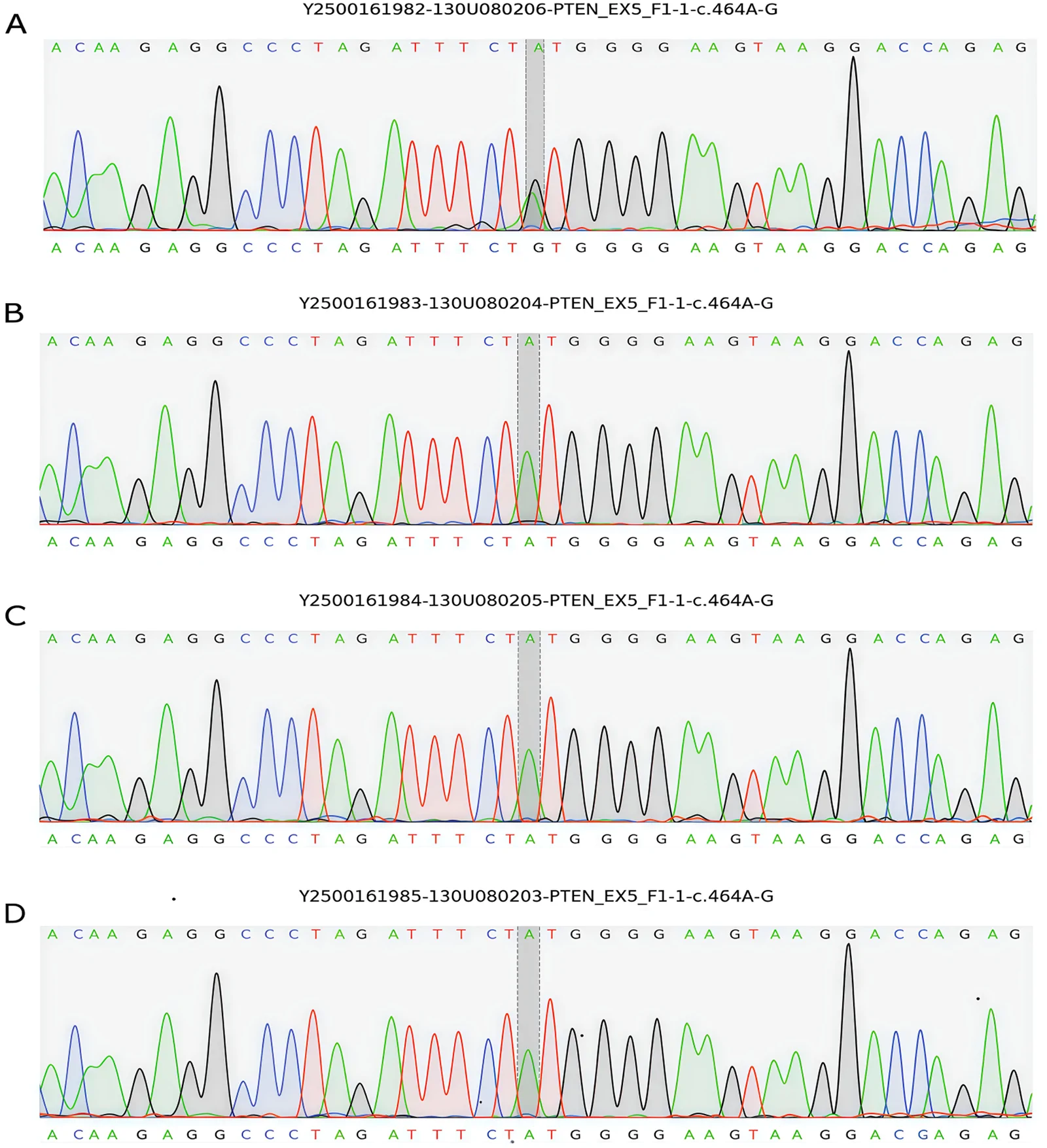
Sanger sequencing validation of PTEN c.464A > G (p.Tyr155Cys). **(A)** Patient **(B)** Patient's father **(C)** Patient's mother **(D)** Patient's older brother.

**Figure 3:**
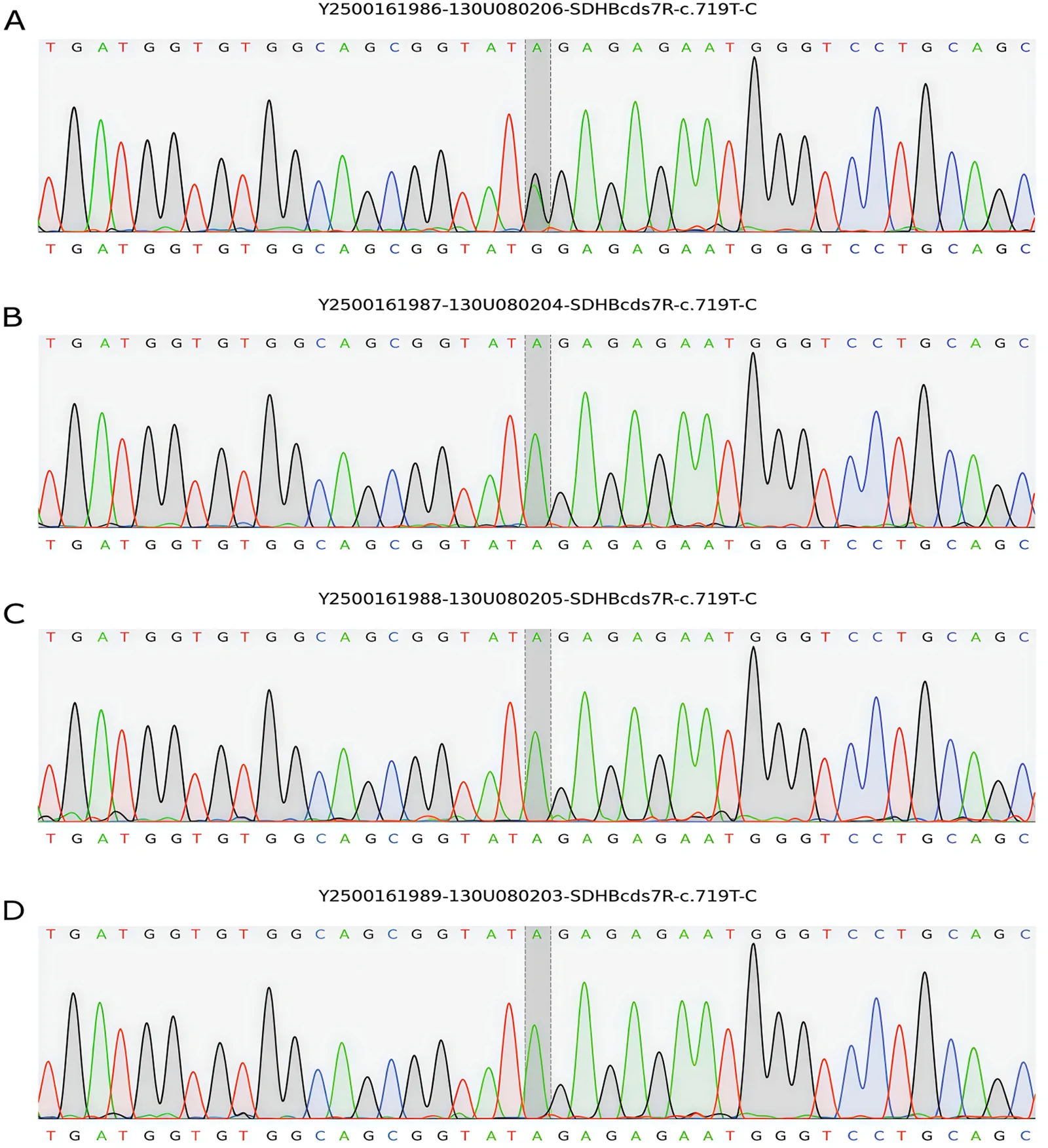
Sanger sequencing validation of SDHB c.719T > C (p.Leu240Pro). **(A)** Patient **(B)** Patient's father **(C)** Patient's mother **(D)** Patient's older brother.

During hospitalization, the patient was diagnosed with gastrointestinal involvement, including a small jejunal polyp identified by capsule endoscopy, mild inflammatory changes involving the distal and terminal ileum, chronic gastritis, duodenitis with erosive lesions, and hypogammaglobulinemia. Histopathological classification of the small bowel polyp was unavailable because tissue sampling was not performed. Her gastrointestinal symptoms improved following corticosteroid therapy. After treatment with corticosteroids, her symptoms got better. To clarify the underlying etiology, family-based whole-exome sequencing was conducted, uncovering a dual heterozygous variants of *PTEN* c.464A > G (p.Tyr155Cys) and *SDHB* c.719T > C (p.Leu240Pro). Both mutations were not present in her parents and older brother, signifying that these variants originated *de novo* in the patient (Figures 2, 3).

Comprehensive evaluation for other PHTS-associated manifestations was also performed. The patient exhibited normal cognitive development without developmental delay or features of autism spectrum disorder. Brain magnetic resonance imaging showed no significant abnormalities of the brain parenchyma. No characteristic mucocutaneous lesions or vascular malformations were identified on physical examination. Thyroid function tests were within normal limits; however, thyroid ultrasonography demonstrated thyroid nodules without evidence of functional thyroid disease. Given the concurrent *SDHB* variant, biochemical surveillance for SDHB-associated tumors was undertaken. Plasma metanephrines and catecholamine metabolite testing were within normal reference ranges, providing no biochemical evidence of catecholamine-secreting tumors. At the 1-year follow-up, no SDHB-related neoplastic manifestations had developed, although the patient continued to experience recurrent upper respiratory tract infections.

A multilevel bioinformatic prediction and structural analysis was performed to assess the possible functional impact of the patient's heterozygous *SDHB* c.719T > C (p.Leu240Pro) variant. Initially, the gnomAD database was used to query the allele frequency of this variant in the general population. The results showed an allele number (AN) of 1,614,026 (GRCh38: chr1:17,022,653T > C) and an allele frequency of 0.000004337. Following the prediction of functional and splicing effects using CADD and SpliceAI, all four metrics produced a CADD score of 7.559 and SpliceAI *Δ* scores of 0. DynaMut2/mCSM was used to perform structural and stability analyses, yielding a *ΔΔ*G of −1.46 kcal/mol. With a buried proline residue and prohibited phi/psi backbone angles, Missense3D categorized the variant as harmful. The Leu240Pro substitution is found within a critical structural domain and decreases residue volume, according to the HOPE platform. A score of 0.974 was assigned by MetaRNN analysis, further supporting the variant's harmful nature.

**Figure 4:**
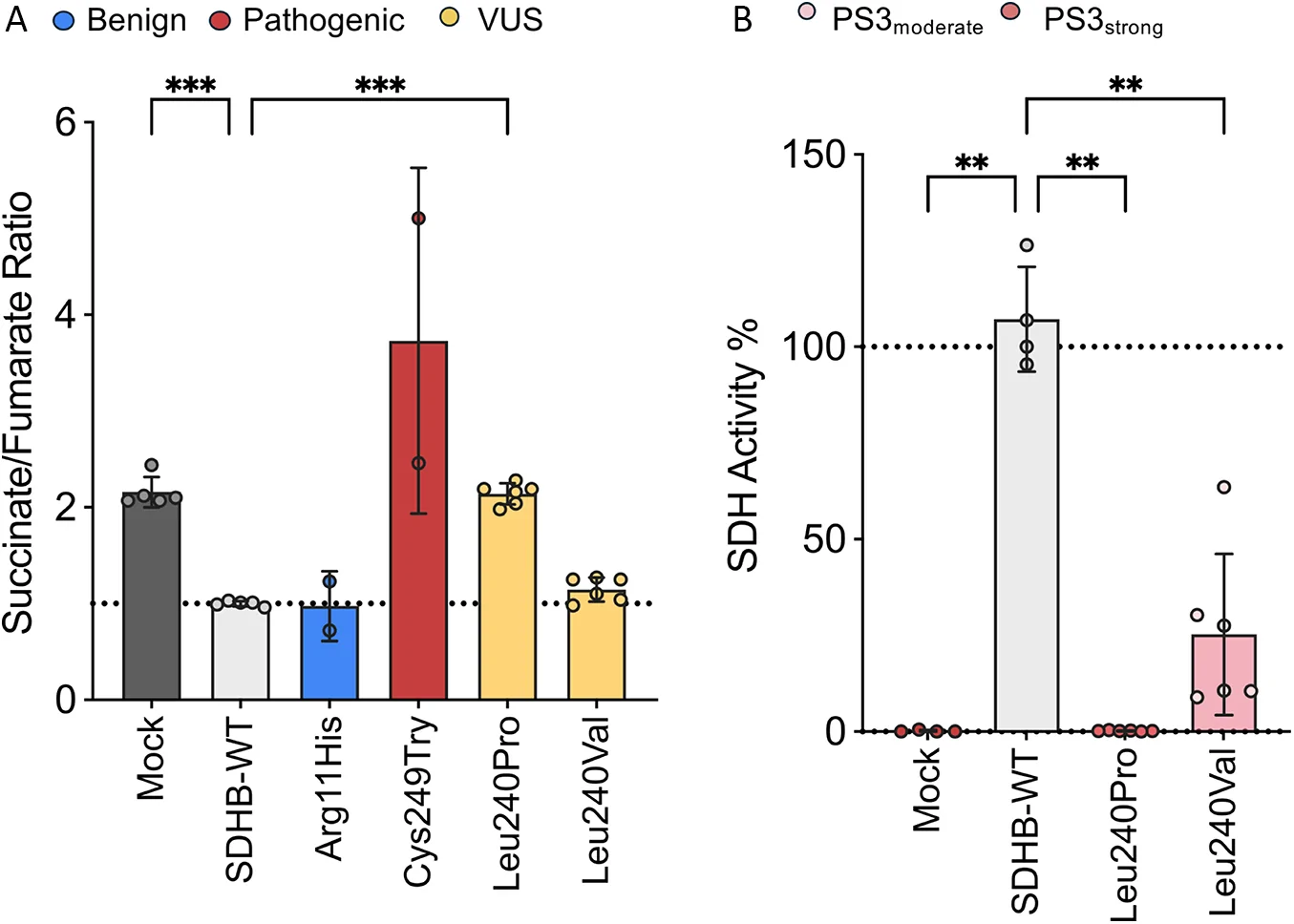
Functional characterization of SDHB variants at residue 240. **(A)** Intracellular succinate/fumarate (S/F) ratio in SDHB-deficient cells expressing WT, benign (Arg11His), pathogenic (Cys249Tyr), and residue 240 variants (Leu240Pro, Leu240Val). **(B)** Corresponding SDH enzymatic activity calculated from S/F ratios.

Next, the *SDHB* c.719T > C (p.Leu240Pro) variant was functionally evaluated using an SDHB-deficient cell complementation system (25). To assess whether residue 240 is broadly intolerant to substitution or specifically sensitive to structural disruption, we included a conservative substitution at the same position (Leu240Val) as a comparator. Cells expressing SDHB wild-type (WT), benign (Arg11His), pathogenic (Cys249Tyr), and the identified Leu240Pro variants were analyzed for intracellular S/F ratio and corresponding SDH activity to assess rescue of mitochondrial complex II function (25). The findings demonstrated that the *SDHB* Leu240Pro variant resulted in a substantial increase in the S/F ratio, indicating a significant reduction in SDH enzyme activity. This pattern indicates hypomorphic activity, is clearly distinct from benign controls, and suggests causative for hereditary pheochromocytoma and paraganglioma syndrome. Conversely, the Leu240Val variant at the same residue exhibited only a mild increase in the S/F ratio and a slight reduction in SDH activity, suggesting preserved function (Figures 4A,B). Moreover, integration of these functional data into the pathogenicity model (25) resulted in a high OddsPath score (55.91) for Leu240Pro, supporting reclassification from a variant of uncertain significance (VUS) to likely pathogenic (Table 1). In contrast, incorporation of functional data had minimal impact on classification of Leu240Val (OddsPath: 7.53), which remained a VUS (Table 1). Together, these results demonstrate that the *SDHB* p.Leu240Pro variant confers a deleterious, loss-of-function phenotype thats aligns with bioinformatic predictions.

**Table 1:** Changes in pathogenicity scores of SDHB p.Leu240Pro and p.Leu240Val variants before and after functional validation.

Variant	ACMG/AMP criteria			Pathogenicity score			
	Population data	Computational data	Functional data	Pre-		Post-	
Leu240Pro	PM2_supporting_	PP3_strong_	PS3_strong_	5	VUS	9	Likely Pathogenic
Leu240Val	PM2_supporting_	Unclassified	PP3_moderate_	1	VUS	3	VUS

“Pre-” interpretations are made from population (BS1/PM2) and computational prediction (BP4/PP3) data. “Post-” interpretations include our functional data (BS3/PS3). Points per strength level: supporting (±1), moderate (±2), strong (±4), no evidence available (Unclassified, 0). Pathogenicity score thresholds: likely benign (LB), between −6 and −1; VUS, between 0 and 5; likely pathogenic (LP), between 6 and 9; pathogenic (P), greater than 10, respectively.

**Figure 5:**
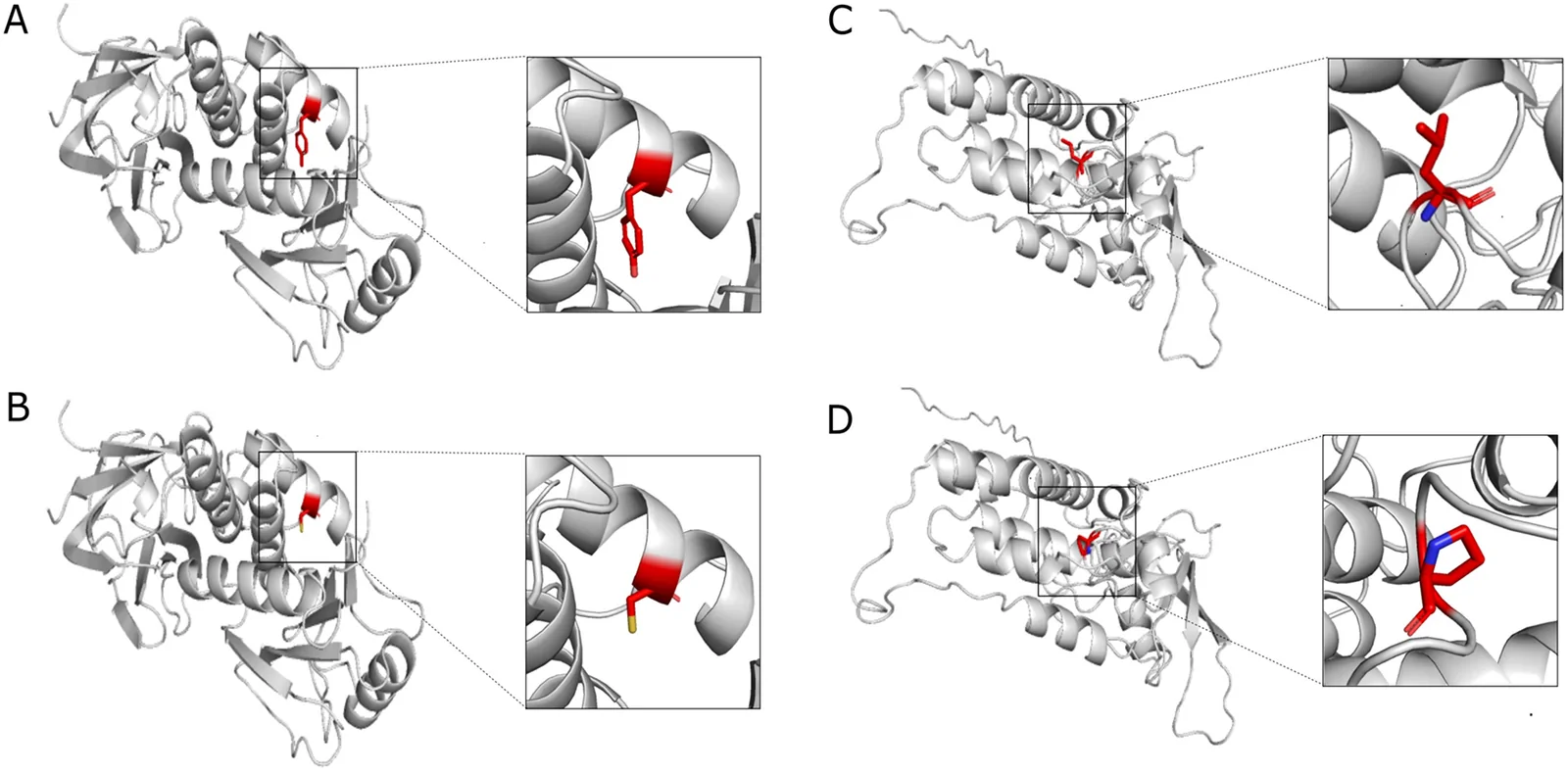
Local conformational changes near PTEN Tyr155 and SDHB Leu240 variant residues. **(A,C)** Predicted 3D structures of wild-type *PTEN* (Tyr155) and *SDHB* (Leu240), respectively. **(B,D)** Predicted structures of mutant *PTEN* p.Tyr155Cys and *SDHB* p.Leu240Pro, respectively.

**Figure 6:**
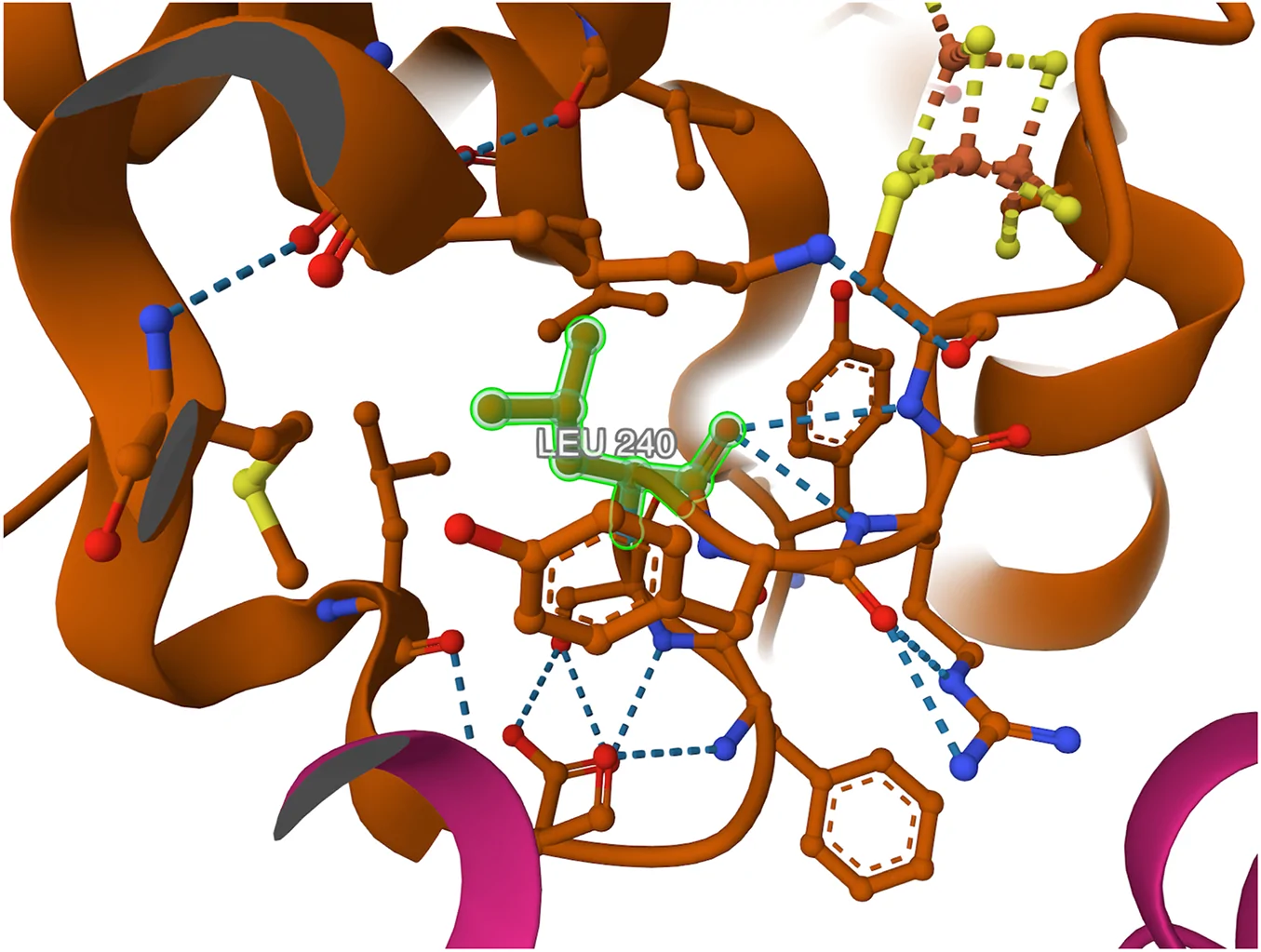
Schematic representation of the SDHB protein structure. Leu240 (highlighted in green) is located near the iron–sulfur (Fe_3_–S_4_) cluster and forms stable hydrogen bond interactions with Cys243 and Arg242. Cys243 coordinates the Fe–S cluster, and Arg242 is part of the conserved LYR motif. These interactions likely contribute to local structural stability within the SDHB subunit.

Furthermore, using PyMOL software, mutant structures for *PTEN* c.464A > G (p.Tyr155Cys) and *SDHB* c.719T > C (p.Leu240Pro) were produced based on protein models of PTEN and SDHB supplied by AlphaFold. Local conformational changes near the variant residues were shown by structural visualization (Figure 5). The schematic representation of the SDHB structure (Figure 6) illustrates that Leu240 is situated in close proximity to the iron-sulfur (Fe–S) cluster and engages in a stable hydrogen bond interaction with the adjacent residues Cys243 and Arg242. Cys243 directly coordinates the Fe–S cluster, while Arg242 is part of the conserved LYR motif, a sequence element found in SDH subunits that is important for Fe–S cluster assembly and stabilization (26). Together, these interactions contribute to the structural integrity of this region. Substitution of leucine with proline, attributable to its distinctive cyclic structure, has the potential to disrupt the local secondary structure and alter the backbone conformation. This disruption may interfere with the spatial arrangement among Leu240, Cys243, and Arg242, thereby compromising the stability of the Fe-S cluster group. Such structural modifications offer a plausible molecular mechanistic explanation for the observed increase in the S/F ratio and the reduction in SDH activity, as evidenced by functional assays.

## 3 Discussion

The patient harbored a heterozygous *PTEN* c.464A > G (p.Tyr155Cys) variant, which is classified as pathogenic in ClinVar and is well established as a cause of PTEN hamartoma tumor syndrome (PHTS). Tyr155 is located within the catalytic core of the PTEN phosphatase domain, and substitution of this residue is predicted to impair phosphatase activity and dysregulate PI3K/AKT signaling (27). Clinically, the patient exhibited macrocephaly, gastrointestinal involvement, thyroid nodules, and immune dysregulation, all of which fall within the recognized phenotypic spectrum of PHTS. Her head circumference (56.5 cm) exceeded the +2 SD upper limit for age-matched females according to WHO and Chinese growth standards (28), consistent with one of the hallmark manifestations of PTEN loss-of-function (29). Taken together, the molecular findings and clinical phenotype suggest that the pathogenic *PTEN* variant represents the primary genetic basis underlying this patient's disease.

In addition to the pathogenic *PTEN* variant, we identified a *de novo* heterozygous *SDHB* c.719T > C (p.Leu240Pro) variant. To the best of our knowledge, this is the first reported *de novo* occurrence of this variant. The allele is extremely rare in the general population (gnomAD allele frequency = 0.000004337) and is currently classified as a variant of uncertain significance (VUS) in ClinVar. To further evaluate its clinical significance, we performed structural modeling, bioinformatic analyses, and functional characterization. Structural modeling based on AlphaFold-predicted structures suggested that substitution of leucine with proline may alter the local conformation surrounding the Fe–S cluster-binding region and may reduce structural stability (25). Functional assays showed an increased intracellular succinate-to-fumarate ratio together with reduced SDH enzymatic activity, findings that are consistent with impaired mitochondrial complex II function. When integrated using the OddsPath framework, these functional data fulfilled ACMG/AMP PS3 evidence and, together with PM2 and PP3 criteria, support our proposed reinterpretation of the variant from VUS to likely pathogenic. Importantly, this proposed reinterpretation is based on our functional evidence, whereas the current ClinVar classification remains VUS.

The coexistence of *de novo* pathogenic *PTEN* and functionally impaired *SDHB* variants in the same individual appears to be extremely uncommon. Although the identification of two *de novo* variants raises the possibility of a more complex genetic background, the patient's major clinical manifestations are consistent with well-recognized features of PHTS and can largely be explained by PTEN deficiency alone. Therefore, the current data do not establish a direct biological interaction between *PTEN* and *SDHB*, nor do they demonstrate that the *SDHB* variant modifies the clinical phenotype beyond that expected for PHTS.

Nevertheless, the functional impairment observed for *SDHB* p.Leu240Pro suggests that this variant is biologically relevant. Previous studies suggest that SDHB dysfunction can influence mitochondrial metabolism and may interact with other tumor-predisposition pathways (30). Accordingly, it remains possible that SDHB-related mitochondrial dysfunction may contributes to phenotypic variability through effects on cellular metabolism or immune homeostasis. However, this possibility should currently be regarded as a hypothesis rather than an established mechanism.

In the present patient, gastrointestinal inflammation, hypogammaglobulinemia, and abnormalities in lymphocyte differentiation were accompanied by clinical improvement following corticosteroid therapy, suggesting an immune-mediated component. Similar immune abnormalities have previously been reported in patients with PHTS (31–34), consistent with the known disease spectrum. Whether SDHB dysfunction contributed to these manifestations cannot be determined from a single case and warrants further mechanistic investigation.

From a clinical perspective, this case highlights the importance of careful long-term surveillance in pediatric patients with atypical multisystem manifestations. The presence of a pathogenic *PTEN* variant is associated with an increased lifetime risk for PHTS-associated neoplasms, including thyroid, breast, endometrial, and gastrointestinal tumors (1, 35). Current guidelines therefore recommend regular tumor surveillance beginning in childhood. In addition, SDHB dysfunction has been associated with paraganglioma, pheochromocytoma, renal cell carcinoma, and gastrointestinal stromal tumors (36–40). Although the pathogenicity of *SDHB* c.719T > C requires further validation, its functional impact suggests that additional monitoring may be warranted. A combined and individualized surveillance strategy may therefore be beneficial for long-term management in this patient. In addition, this case highlights the importance of comprehensive genetic counseling and consideration of prenatal or preimplantation genetic testing in families affected by rare disease-associated variants, particularly when complex or atypical phenotypes are present.

Several limitations should also be acknowledged. First, this study describes a single case, and the phenotypic contribution of the *SDHB* variant cannot be definitively established. Second, although structural prediction and functional analyses support the potential pathogenicity of *SDHB* c.719T > C, the precise mechanism underlying the potential interaction between *PTEN* and *SDHB* remains unresolved. Further experimental studies will be necessary to clarify whether SDHB dysfunction may act as a phenotypic modifier in PTEN-related disease.

In conclusion, we report a pediatric patient harboring a pathogenic *PTEN* c.464A > G (p.Tyr155Cys) variant together with a functionally characterized *SDHB* c.719T > C (p.Leu240Pro) variant for which our data support a likely pathogenic interpretation. To our knowledge, this is the first reported *de novo* occurrence of the SDHB variant and also the first report describing coexisting *PTEN* and *SDHB* variants in a patient with this phenotype. The patient presented with macrocephaly, gastrointestinal involvement, and immunological abnormalities, consistent with a multisystem presentation. This case expands the molecular and clinical spectrum associated with PTEN-related disorders and suggests that potential multigenic contributions should be considered in patients with complex phenotypes. Early recognition and long-term follow-up remain essential for individualized clinical management.

## 4 Methods

### 4.1 Functional characterization of SDHB variants

Functional assessment of SDHB variants was performed using a previously established complementation assay in SDHB-knockout cells, as described in (25). Briefly, SDHB variant constructs were transiently expressed in SDHB-KO cells (48 h), and intracellular succinate and fumarate levels were measured by targeted LC–MS/MS using a UHPLC–triple quadrupole mass spectrometry platform (Agilent). Succinate-to-fumarate ratios were used to calculate relative SDH enzymatic activity, with values normalized to wild-type (100%) and knockout controls (∼0%) within each experiment. This approach enabled consistent comparison across experiments and variants.

### 4.2 SDHB variant pathogenicity interpretation

Variant pathogenicity was then inferred using a logistic regression model trained on known benign and pathogenic *SDHB* variants, as previously described (25). The model generates a probability of pathogenicity [P(path)], which was converted to OddsPath [p/(1 − p)] and used to assign ACMG/AMP functional evidence strengths (PS3/BS3).

### 4.3 Structural modeling of SDHB variants

Protein structural coordinates for *SDHB* variants were obtained from the RCSB Protein Data Bank (PDB ID: 8GS8).

## Data Availability

The original contributions presented in the study are included in the article/Supplementary Material, further inquiries can be directed to the corresponding author/s.
