# Correction: Coexisting PTEN and SDHB mutations in a pediatric patient with PTEN hamartoma tumor syndrome: a case report

**DOI:** 10.3389/fped.2026.1955287

**Published:** 2026-08-25

**Authors:** Yaqin Zhang, Sooyeon Lee, Justin P. Annes, Jie Zhang, Taicheng Zhou, Yuan Qian, Xingli Deng

**Affiliations:** 1 Department of Reproductive Genetics, Pu'er People’s Hospital, Pu'er, China; 2 Department of Medicine, Division of Endocrinology, Stanford University, Stanford, CA, United States; 3 Stanford ChEM-H and Endocrine Oncology, Stanford University Cancer Institute, Stanford, CA, United States; 4 Genetics Department, The First People’s Hospital of Yunnan Province, Kunming, China; 5 Central Lab, Clinical Diagnosis and Treatment Center for Precision Medicine, The Affiliated Hospital of Yunnan University/Second People’s Hospital of Yunnan Province, Kunming, China; 6 Department of Neurosurgery, The First Affiliated Hospital of Kunming Medical University, Kunming, China

**Keywords:** macrocephaly, multigenic interaction, precision medicine, PTEN hamartoma tumor syndrome, *PTEN mutation*, *SDHB variant*

The order of authors in the author list of the published paper was erroneously given as:

Yaqin Zhang, Sooyeon Lee, Justin P. Annes, Jie Zhang, Taicheng Zhou, Xingli Deng* and Yuan Qian*

The correct author list reads:

Yaqin Zhang, Sooyeon Lee, Justin P. Annes, Jie Zhang, Taicheng Zhou, Yuan Qian* and Xingli Deng*

The original version of this article has been updated.

